# The RESCUE Technique: A Mnemonic Acronym to Enhance Outcomes in Nail Fixation of Extracapsular Hip Fractures

**DOI:** 10.3390/jcm14155419

**Published:** 2025-08-01

**Authors:** Anastasios P. Nikolaides, Julius Bryan Abesamis, Ahmed Hamed, Samer Sarofeen, Niraj Vetharajan, Rajpreet Sahemey, Omer Salar, Panagiotis Konstantinou

**Affiliations:** Orthopaedic Department, University Hospitals Birmingham NHS Foundation Trust, Birmingham B7 5TE, UK; anastasios.nikolaidis@nhs.net (A.P.N.); jcabesamis1@up.edu.ph (J.B.A.); ahmed.hamed@nhs.net (A.H.); samerhsarofeen@gmail.com (S.S.); niraj.vetharajan@uhb.nhs.uk (N.V.); rajpreet.s@gmail.com (R.S.); omer.salar@uhb.nhs.uk (O.S.)

**Keywords:** cephalomedullary nail, intertrochanteric fractures, RESCUE technique, screw placement, fracture reduction, implant failure

## Abstract

Intertrochanteric fractures in the elderly present complex challenges due to poor bone quality and comorbidities. Cephalomedullary (CM) nails offer biomechanical advantages that support early mobilization, yet complications such as cutout, implant failure, and malalignment persist. This review examines the effectiveness of CM nail fixation in geriatric extracapsular hip fractures and introduces the **RESCUE** technique—a structured, mnemonic-based approach aimed at improving surgical outcomes and reducing common complications. **RESCUE** stands for Reduce, Entry point, Screw, Compress, Unleash traction, and Enhance full-weight bearing. This six-step framework addresses the critical elements of fixation, including precise reduction, optimal entry point selection, central screw placement, controlled fracture compression, cautious traction management, and early mobilization. Case illustrations of frequent failure patterns underscore the practical application of the RESCUE technique. By following this systematic approach, surgeons can enhance construct stability, minimize failure risk, and promote functional recovery in elderly patients.

## 1. Introduction

Intertrochanteric fractures in older adults, especially those with unstable patterns such as AO/OTA (Arbeitsgemeinschaft für Osteosynthesefragen/Orthopaedic Trauma Association) 31A3, are associated with significant morbidity [1,2]. These fractures often fail to share the weight-bearing load adequately, with the fixation device bearing much of the stress. The four classic fracture patterns that are commonly linked to instability include: reverse obliquity fractures, transtrochanteric fractures, fractures with a large posteromedial fragment (indicating loss of the calcar buttress), and fractures extending into the subtrochanteric region [3]. Cephalomedullary (CM) nails are often the preferred treatment for these fractures due to their biomechanical advantages, including facilitating early postoperative mobilization. However, stable fixation in osteoporotic bone is challenging, with potential complications such as screw cutout and implant failure often linked to technical issues.

Unstable intertrochanteric fractures carry a high failure risk when treated nonoperatively or with extramedullary implants. In contrast, numerous studies support the use of intramedullary devices, particularly cephalomedullary (CM) nails, as a more effective option for internal fixation. CM nails provide superior load-sharing in osteoporotic bone, promoting fracture healing, facilitating early mobilization, and allowing earlier weight-bearing—key factors that enhance functional recovery and reduce morbidity, especially in elderly patients [4,5,6]. Clinical studies consistently report high union rates, favorable functional outcomes, and fewer complications with CM nails, particularly when anatomical reduction and proper screw positioning are achieved. Moreover, CM nails are associated with lower mortality rates, faster rehabilitation, and improved overall outcomes compared to alternative treatments, such as extramedullary fixation, which is often linked to higher failure rates and poorer functional recovery [7]. However, other studies have found no significant differences in clinical outcomes between intramedullary nails and extramedullary devices, emphasizing the need for further research to clarify the comparative effectiveness of these treatment strategies [8].

Importantly, technical precision—especially regarding entry point, fracture reduction, and screw placement—is essential to avoid fixation failure. Despite the clear advantages, CM nail fixation requires meticulous technique to avoid failure. A poor entry point, inadequate fracture reduction, or improper screw placement can lead to complications, highlighting the need for careful surgical planning.

Despite their advantages, CM nail fixation is associated with complications such as screw cutout, varus malalignment, and femoral shaft fractures, with screw cutout rates reported between 5% and 16% [9,10]. These issues often arise from suboptimal entry point, inadequate reduction, or improper screw placement. Understanding common failure mechanisms—such as varus collapse, femoral shaft medialization, and hardware failure—can help guide more effective nail placement and fracture reduction. This technical note discusses CM nail use for fragility intertrochanteric fractures and introduces the RESCUE technique—a structured mnemonic to improve fixation stability and minimize complications.

## 2. Introduction of the RESCUE Technique

**Scheme 1:**
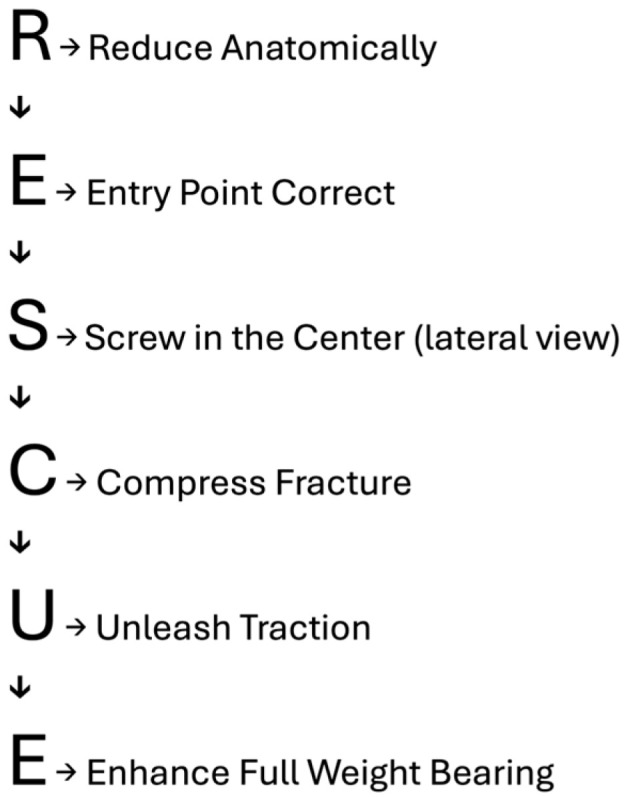
Visual flowchart of the steps of the RESCUE technique.

The RESCUE mnemonic was developed to address common pitfalls in CM nail fixation and reduce the risk of cutout in fragile intertrochanteric fractures (Scheme 1). The key steps of this technique are outlined below:

### 2.1. R—Reduce Anatomically

Achieving an anatomical reduction is critical for ensuring stable fixation in unstable intertrochanteric fractures. Lateral and anteroposterior (AP) imaging should be used to confirm alignment, as any misalignment during reduction will persist once the nail is inserted. Varus angulation of the proximal fragment increases the mechanical stress on the fixation device, raising the risk of screw cutout [11]. Proper reduction ensures the femoral head is aligned, and the fixation device is optimally placed [12].

**Figure 1:**
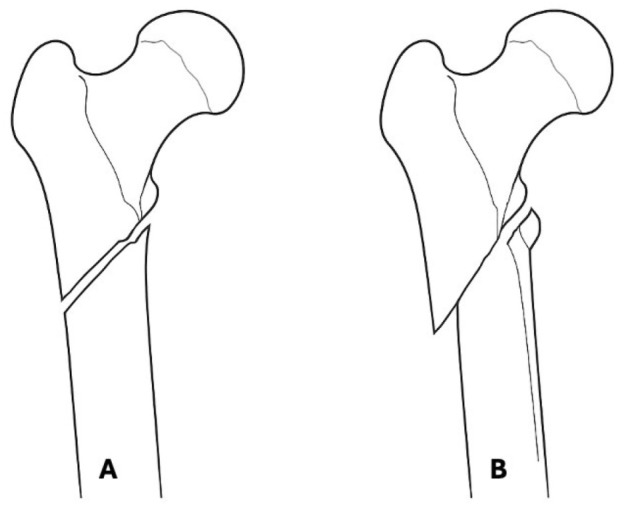
Types of medial cortex support post reduction: (**A**). Positive medial cortex support. (**B**). Negative medial cortex support.

Technical Tip: Achieving Positive Medial Cortex Support (PMCS) is critical for fracture stability. As described by Chang et al. [13], in this configuration, the proximal femoral head–neck fragment is medially displaced to align with the upper medial edge of the distal femoral shaft. In Negative Medial Cortical Support (NMCS), the medial cortex of the proximal fragment lies lateral to or is not in contact with the medial cortex of the distal fragment (Figure 1). This restoration of medial cortex support enhances load distribution, improves mechanical stability, and minimizes the risk of angular deformities during the healing process. PMCS has also been associated with improved postoperative outcomes, including lower complication rates and better patient recovery, according to a recent study [14].

### 2.2. E—Entry Point Correct

Ensuring the correct entry point for nail insertion is essential to avoid malalignment and achieve proper fracture fixation. The optimal entry point is at the tip of the greater trochanter, aligned with the femoral shaft. This position minimizes the risk of malrotation and improper alignment during insertion. However, one challenge is the tendency of the pilot hole to enlarge laterally during reaming and nail insertion, which can lead to a more lateral nail position than intended. To counteract this, positioning the entry point slightly medial to the tip of the greater trochanter is recommended. This adjustment helps prevent lateral enlargement of the pilot hole, ensuring correct alignment of the nail [3]. The use of a flexible reamer can mitigate inaccuracies in the entry point, particularly in obese patients, by facilitating a more medial entry point [15].

### 2.3. S—Screw in Center (Lateral View)

The positioning of the lag screw centrally within the femoral head on the lateral view is critical for maximizing screw holding strength and enhancing fixation stability. On the anteroposterior (AP) view, a central or slightly inferior placement of the lag screw is considered optimal [12]. Furthermore, central placement of the screw on the lateral view has been associated with lower rates of implant-related complications (IRCs), according to a recent study [16]. Ensuring a Tip-Apex Distance (TAD) of less than 25 mm is essential for minimizing the risk of screw cutout [16]. Traditional techniques that placed the lag screw inferiorly and posteriorly resulted in an increased TAD, raising the risk of failure. Recent recommendations suggest positioning the screw centrally and 10 mm short of the subchondral bone to optimize fixation [3,17]. In addition, the calcar-referenced tip-apex distance (CalTAD) also appears to play a significant role in predicting implant failure in geriatric intertrochanteric fractures, according to recent studies [18,19].

### 2.4. C—Compress Fracture

Applying controlled compression across the fracture site is essential for minimizing micromotion and promoting healing. While interlocking screws designed to create compression are often used, they may fail to engage properly in some cases [20]. Surgeons should confirm the correct engagement of the compression mechanism or consider alternative methods to ensure adequate compression and minimize the risk of nonunion. Compression acts like pre-sliding prevent the postoperative shortening of the femoral neck [21]. According to a recent study, the twin-screw integrated cephalomedullary (CM) nail is clinically more effective than the single-screw proximal femoral nail antirotation (PFNA) in terms of reduced implant-related failures, revision rates, and postoperative pain [22].

Calcar fracture gapping is a crucial determinant in evaluating the quality of fracture reduction. Baumgaertner et al. [17] demonstrated that fragment displacement exceeding 4 mm in either the AP or lateral view is considered unacceptable, as it can adversely affect the stability and success of fixation. Likewise, Lobo-Escolar et al. [23] reported that postoperative fragment diastasis greater than 3 mm is significantly associated with cut-out complications in patients treated with femoral intramedullary nailing.

The presence of a larger calcar gap may contribute to the loss of anteromedial cortical support during the sliding process, as suggested by Chang et al. [24]. In addition to calcar fracture gapping, several other factors may influence the sliding mechanism and compromise anteromedial cortical support. These factors include the capacity to activate head-neck sliding, the orientation of the sliding trajectory, external rotation of the femoral shaft, and rotational movements during sliding [25]. Collectively, these variables highlight the complex interplay of factors that impact fracture healing and fixation stability, underscoring the importance of meticulous management to ensure optimal outcomes in femoral fracture treatment.

### 2.5. U—Unleash Traction

Gradually releasing traction during fracture fixation reduces stress on the fixation device and helps prevent over-distraction. Excessive traction can compromise fracture alignment, increasing the risk of malunion or implant failure [26].

Once traction is unleased, adequate compression should be applied to the fracture on the table to ensure optimal reduction. The fracture should be sufficiently compressed to prevent collapse, which supports proper union post-fixation. Failure to release traction prior to the final tightening of the neck screws can result in screw back-out, which may be evident on immediate postoperative imaging [26].

### 2.6. E—Enhance Full-Weight Bearing

Encouraging early full weight bearing is tolerated to promote bone healing and prevent muscle wasting. Early mobilization is particularly critical in elderly patients to reduce the 30-day mortality rate and the post-op complications rate [6]. Research shows that early mobilization is one of the most effective strategies to reduce morbidity and mortality, facilitating recovery, and restoring pre-fracture function. Prompt surgical intervention, combined with early mobilization, improves outcomes and reduces the risk of pressure ulcers [27,28]. In a recent study involving 410 geriatric patients with intertrochanteric fractures treated with cephalomedullary nails, immediate weight-bearing was shown to improve early functional outcomes without increasing mortality or complication rates [29].

### 2.7. Case Examples

#### 2.7.1. Case 1—Failure to Reduce Anatomically

Learning Point: The anatomical reduction RESCUE step was omitted, which led to the loss of medial cortex support, varus alignment, and fixation failure, ultimately requiring a revision operation two months postoperatively.

**Figure 2:**
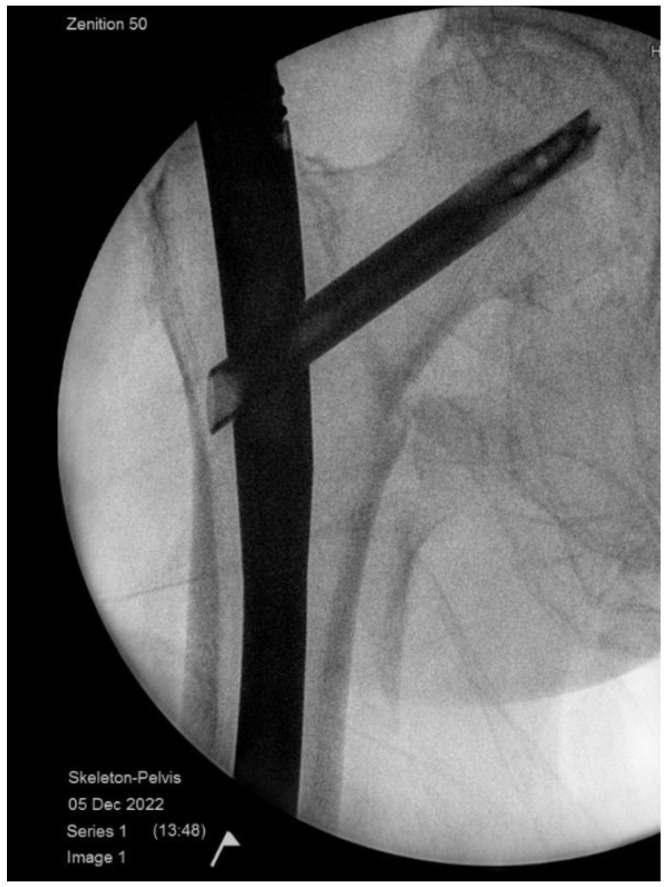
Varus reduction in intertrochanteric fracture. The head–neck fragment is displaced laterally, resulting in the loss of medial cortex support from the femoral shaft.

**Figure 3:**
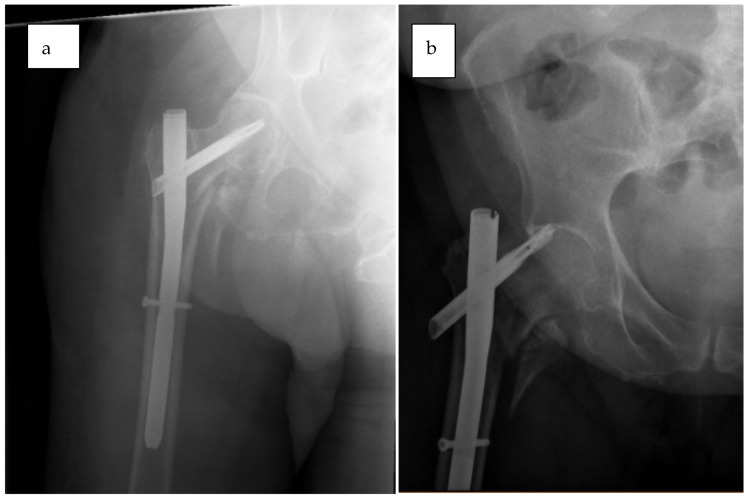
Immediate Postoperative Radiograph (**a**) vs. 8-Week Follow-Up Radiograph of the Hip (**b**).

An intertrochanteric fracture reduced in varus led to medial cortex support loss and implant cutout, necessitating revision surgery to restore alignment (Figure 2). Postoperative radiographs revealed significant cutout due to negative medial cortex support (Figure 3), indicating the need for improved anatomical reduction and valgus alignment to prevent failure.

In this case, the fracture was reduced in varus, with the head–neck fragment displaced laterally. This misalignment resulted in the loss of medial cortex support from the femoral shaft. Achieving an anatomically accurate or slightly valgus reduction prior to definitive fixation, particularly when using intramedullary nailing (CMN), is crucial for improving stability. A valgus or even positive reduction, where the fracture is angulated outward, can enhance load bearing across the joint and improve overall mechanical stability.

As described by Chang et al. [13], Negative Medial Cortex Support (NMCS) occurs when the head–neck fragment is displaced laterally to the upper medial edge of the shaft fragment, resulting in the loss of medial cortex support from the femoral shaft. This lack of support compromises fracture stability and increases the risk of mechanical failure or nonunion.

In contrast, achieving Positive Medial Cortex Support (PMCS), where the proximal femoral head–neck fragment is displaced medially to align with the upper medial edge of the distal femoral shaft fragment, provides better stability. This restoration of medial cortex support enhances load distribution and reduces the risk of angular deformities during healing.

At 8 weeks postoperatively, the follow-up radiograph (B) revealed significant implant cut-out, indicating failure of fixation. This failure was likely due to negative medial cortex support, failure to achieve valgus alignment, or inadequate anatomical reduction during the initial surgery. As a result, revision surgery was necessary, which included the removal of the failed implant and conversion to hip replacement.

#### 2.7.2. Case 2—Unable to Unleash Traction

Learning Point: The “unleash traction” RESCUE step was omitted, which led to an increased fracture gap and an unstable construct, resulting in fixation failure.

Intraoperative radiographs showed unreleased traction before final fixation, resulting in a distracted and unstable construct. Postoperative images at two months showed failure due to insufficient compression and improper reduction. Full traction release before neck screw tightening is essential to prevent screw back-out and ensure stable fixation [26].

**Figure 4:**
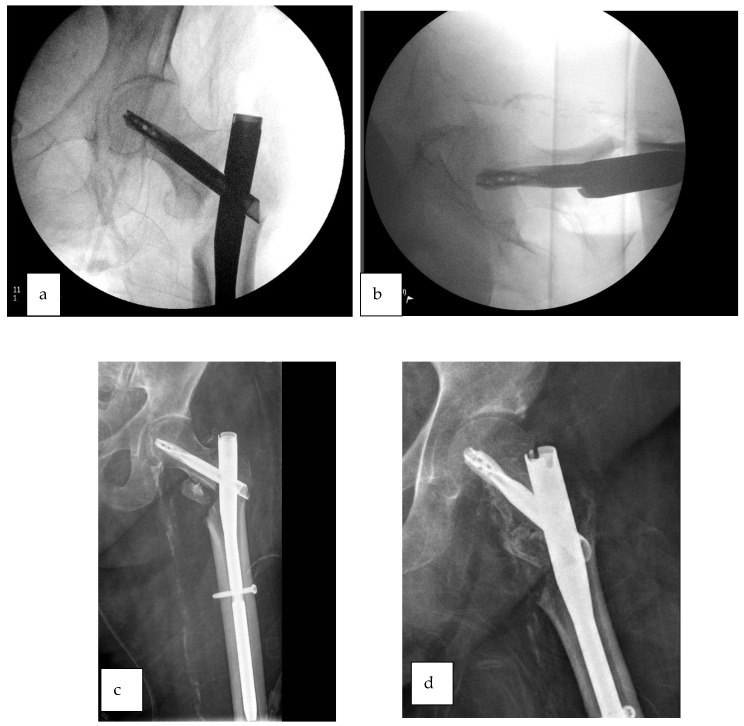
(**a**,**b**) Intraoperative AP and Lateral Hip Radiographs Showing Unreleased Traction, (**c**,**d**) at 2 Months Demonstrating Distracted and Unstable Construct.

In this case, intraoperative anteroposterior (AP) and lateral radiographs of the hip (Figure 4a,b) revealed that traction was not fully released before final fixation. As a result, the construct remained distracted, with a large calcar gap, leading to an unstable configuration. Postoperative radiographs taken two months later (Figure 4c,d) demonstrated the distracted and unstable construct, indicating that optimal reduction and compression were not achieved during the initial surgery. The failure to fully release traction prior to tightening the screws compromised fracture stability, which ultimately led to implant failure. This necessitated revision surgery to restore anatomical alignment, achieve proper compression, and facilitate successful bone healing.

## 3. Discussion

Unstable intertrochanteric fractures in elderly patients pose significant challenges due to high morbidity and mortality, often resulting from complications such as implant cut-out, malalignment, and delayed union. Cephalomedullary (CM) nails are the preferred treatment, particularly in osteoporotic bone, due to their biomechanical advantages, including enhanced load distribution and support for early mobilization. However, the risk of complications, such as cut-out, misalignment, and femoral shaft fractures, highlights the need for technical precision during surgery.

The RESCUE technique offers a structured, evidence-based approach to optimize CM nail fixation outcomes. This six-step framework—Reduce Anatomically, Entry Point Correct, Screw in Center, Compress Fracture, Unleash Traction, and Enhance Full-Weight Bearing—addresses common pitfalls in intramedullary nailing. By focusing on precise reduction, accurate entry point selection, central screw placement, controlled compression, gradual traction release, and early mobilization, RESCUE enhances fracture stability, reduces nonunion, implant failure, and malalignment, and improves overall healing outcomes in elderly patients with intertrochanteric fractures.

Proper reduction prior to nail insertion is critical to avoid varus deformity. A positive medial cortex or valgus reduction technique has been shown to provide greater stability and improve the potential for union. Chang et al. [13] demonstrated that positive medial cortical support—a nonanatomic reduction method placing the medial cortex of the proximal fragment superomedial to that of the distal fragment—results in excellent radiological outcomes.

The cut-out of the PFNA blade from the femoral head is a severe complication, especially in frail elderly patients. Yam et al. [30] found that maintaining a tip-apex distance of less than 27 mm and a neck-shaft angle greater than 128° is essential in preventing this complication. Additionally, calcar referenced tip-apex distance (CalTAD) improves upon traditional TAD by using a line parallel to the femoral neck adjacent to the calcar, instead of the femoral neck’s center, promoting more inferior-central lag screw placement and potentially improving fixation stability [18,19,31].

Optimal blade placement is critical, with many authors recommending center-center or inferior-center positioning [16]. Yam et al. [30] highlighted that posterior and superior placements increase the risk of cut-out, further emphasizing the importance of accurate blade positioning. In patients with AO 31A3 unstable fractures, heightened awareness of cut-out complications is essential. Regular follow-up can facilitate early detection, enabling timely intervention before joint perforation occurs [30].

Limited fracture gapping is advantageous for facilitating fragment sliding and enhancing secondary stability, promoting medial cortex-to-cortex contact. However, this buttressing effect is compromised if the gap exceeds the thickness of a single cortical layer. Due to the biomechanical properties of the hip joint, the head-neck fragment slides obliquely in the inferior-lateral direction until it contacts the intramedullary nail, which provides additional implant support [25].

Finally, studies underscore the importance of avoiding varus reduction, which leads to unstable fixation and higher rates of cut-out in intertrochanteric fractures [32,33,34]. Therefore, incorporating the RESCUE technique, with its focus on precise reduction and optimal implant positioning, can significantly reduce cut-out rates and improve overall outcomes, particularly for elderly patients who are at an elevated risk for these complications.

The RESCUE technique provides a systematic framework for improving the surgical management of unstable intertrochanteric femoral fractures, particularly in elderly patients with osteoporotic bone. It is especially useful in complex fracture patterns—such as those involving comminution or reverse obliquity—where cephalomedullary nailing is the preferred method of fixation. By emphasizing key technical steps, including accurate reduction, optimal entry point selection, central positioning of the lag screw, and proper compression, the technique helps support earlier mobilization and enhances the likelihood of successful healing.

Nevertheless, its application requires careful attention to detail and intraoperative imaging, making it more technically demanding than standard approaches. It may not be appropriate for patients with stable fracture configurations, significant comorbidities precluding surgery, or anatomical challenges that prevent safe nail insertion. For these reasons, thoughtful case selection and meticulous surgical execution are essential to achieving the full benefits of the RESCUE strategy while minimizing potential complications.

Further research is warranted to validate the findings and evaluate the long-term outcomes of this rescue technique. A retrospective cohort study examining postoperative results in patients with fragility intertrochanteric fractures treated using this method could provide valuable insight into its clinical effectiveness and potential limitations. Additionally, a prospective comparative study between the rescue technique and conventional nailing methods would help establish its relative advantages in terms of complication rates, functional outcomes, and implant survival. Such studies could contribute significantly to optimizing surgical strategies for this challenging patient population. Future studies could enhance postoperative outcomes in these fractures by enabling the design and use of patient-specific femoral implants [35].

## 4. Conclusions

The RESCUE technique offers a structured and thoughtful approach to the management of intertrochanteric femur fractures, emphasizing principles known to influence surgical success. While preliminary observations suggest its potential to reduce complications and support timely recovery, further clinical validation—through retrospective and prospective studies—is needed. With consistent application and continued evaluation, this technique may contribute meaningfully to improved outcomes in the treatment of fragility fractures.

## Data Availability

Data sharing is not applicable.
